# *Plasmodium falciparum kelch13* polymorphisms identified after treatment failure with artemisinin-based combination therapy in Niger

**DOI:** 10.1186/s12936-023-04571-w

**Published:** 2023-05-01

**Authors:** Ibrahima Issa Arzika, Neil F. Lobo, Mahaman Moustapha Lamine, Ilagouma Amadou Tidjani, Houzé Sandrine, Véronique Sarrasin-Hubert, Aboubacar Mahamadou, Eric Adehossi, Demba Sarr, Ousman Mahmud, Ibrahim Maman Laminou

**Affiliations:** 1 Centre de Recherche Médicale et Sanitaire de Niamey, Niamey, Niger; 2 University of Notre Dame, Notre Dame, USA; 3 Université André Salifou de Zinder, Zinder, Niger; 4 University Abdou Moumouni of Niamey, Niamey, Niger; 5 University of Georgia, Athens, USA; 6 Centre National de Référence du Paludisme, Paris, France

**Keywords:** *P. falciparum*, Resistance, Artemisinin, *Pfkelch*13, Niger

## Abstract

**Background:**

Artemisinin-based combination therapy (ACT) is the most effective treatment for malaria, and has significantly reduced morbimortality. Polymorphisms associated with the *Plasmodium falciparum* Kelch gene (*Pfkelch13*) have been associated with delayed parasite clearance even with ACT treatment.

**Methods:**

The *Pfkelch13* gene was sequenced from *P. falciparum* infected patients (n = 159) with uncomplicated malaria in Niger. An adequate clinical and parasitological response (ACPR) was reported in 155 patients. Four (n = 4) patients had treatment failure (TF) that were not reinfections—two of which had late parasitological failures (LPF) and two had late clinical failures (LCF).

**Results:**

Thirteen single nucleotide polymorphisms (SNPs) were identified of which seven were non-synonymous (C469R, T508S, R515T, A578S, I465V, I437V, F506L,), and three were synonymous (P443P, P715P, L514L). Three SNP (C469R, F506L, P715P) were present before ACT treatment, while seven mutations (C469R, T508S, R515T, L514L, P443P, I437V, I465V) were selected by artemether/lumefantrine (AL)—five of which were non-synonymous (C469R, T508S, R515T, I437V, I465V). Artesunate/amodiaquine (ASAQ) has selected any mutation. One sample presented three cumulatively non-synonymous SNPs—C469R, T508S, R515T.

**Conclusions:**

This study demonstrates intra-host selection of *Pfkelch13* gene by AL. The study highlights the importance of LCF and LPF parasites in the selection of resistance to ACT. Further studies using gene editing are required to confirm the potential implication of resistance to ACT with the most common R515T and T508S mutations. It would also be important to elucidate the role of cumulative mutations.

## Background

The use of artemisinin-based combination therapy (ACT)—combining a derivative of artemisinin (dihydroartemisinin, artesunate, or artemether) with a partner molecule (amodiaquine, lumefantrine, pyronaridine, mefloquine, piperaquine, or sulfadoxine-pyrimethamine), has led to a significant reduction in malaria morbidity and mortality in tropical and subtropical areas globally [1].

However, the emergence of *Plasmodium falciparum* strains resistant to artemisinin is a major challenge threatening the viability of current global operations intended to reduce the burden of malaria [2, 3]. Indeed, the sensitivity of *P. falciparum* strains to ACT has been decreasing steadily in Southeast Asia since 2008. Resistance to artemisinin first emerged in Cambodia and then spread across countries, such as Thailand, Myanmar and Vietnam in the Mekong basin [4]. Similar to the widespread dissemination of resistance to chloroquine and sulfadoxine-pyrimethamine, the spread of artemisinin resistance should be expected and remains a global concern [5].

Artemisinin-based resistance usually manifests clinically as an increase in the parasite clearance time post treatment. Thus, late parasitological failures (LPF) (where parasitaemia reappears without symptoms, from day 7 to 28 post treatment), and early and even late clinical failures (LCF) (where symptoms develop during the follow-up period from day 4 to 28 post treatment), are increasingly observed in therapeutic efficacy studies [6]. This resistance to artemisinin is no longer confined to Southeast Asia and the first cases of artemisinin failure in Africa have been observed in Rwanda [7, 8] and Uganda [9].

The complete sequencing of the *Plasmodium* genome along with the emergence of ACT resistance led to the 2014 discovery of the *Pfkelch13* (*k13*) gene [10] located on chromosome 13—polymorphisms within which are strongly associated with artemisinin resistance both in vitro and in vivo. This *k13* marker allows both the monitoring of the emergence and spread of characterized and novel alleles, as well as geographic mapping of resistance to artemisinin [11].

Several studies have been conducted around the world (59 countries, 163 sites) towards characterizing polymorphisms in this gene and assessing the predictive value of a point mutation to therapeutic failure with artemisinin. These studies revealed that this gene is highly polymorphic with 108-point mutations highlighted in the KARMA study (K13 Artemisinin Resistance Multicenter Assessment). Nine mutations were described as being both the most important and associated with resistance: C580Y, F446I, R539T, A578S, Y493H, P574L, P553L, N458Y and R561H [12]. However, A578S was later confirmed by gene editing to be not an artemisinin resistance mutation [12].

In Africa, ACT efficacy begins to fail [13]. A multicentre study was conducted on the K13 gene in Sahel countries. Twenty-two mutations were identified, of which seven non-synonymous mutations were highlighted—the most important being A557S, V566I, A569T, S576L,, L589I, Y630F, A578S [14]. This first comprehensive and systematic review focusing on polymorphisms of the *k13* gene provides a valuable baseline reference for building and strengthening surveillance activities in Africa [15].

In Niger, two main studies were conducted on this gene. One study revealed thirteen-point mutations. Six out of these thirteen-point mutations were non-synonymous and seven were synonymous. Eight mutations were specific to Niger with five being non-synonymous [16]. Another study evaluated the association between *k13* gene mutations and response to treatment with artemether-lumefantrine (AL, Coartem^®^) and artesunate-amodiaquine (ASAQ, Winthrop^®^) in Gaya, Niger. The nature of single nucleotide polymorphisms (SNPs) before and after treatment were analysed as evidence of resistance selection following drug pressure. Five-point mutations were found with three being non-synonymous and two synonymous. Four of these mutations were observed prior to ACT treatment. Only one non-synonymous mutation was selected for by ASAQ: *k13* A569G [16].

However, the prevalence and role of *k13* mutations are still poorly understood [17]. The major question that remains is ‘what is the relationship between *k13* alleles and therapeutic failure with artemisinin or parasite clearance?’.

This study was conducted towards characterizing *k13* point mutations and their relationship with therapeutic failure with artemisinin. Sequencing data presented herein highlights polymorphisms in the *k13* gene and their association with treatment failure with AL or ASAQ in Niger.

## Methods

### Study design

This is a descriptive and analytical study of *k13* gene polymorphisms using isolates from a randomized, two-armed clinical trial comparing AL versus ASAQ. This study analysed the association between selected mutations and anti-malarial drugs (AL or ASAQ) as well as the association between selected mutations and treatment failure (LCF, LPF).

### Study site

This study was conducted in Dogondoutchi, a sentinel site of the National Malaria Control Programme (NMCP), Niger. Dogondoutchi is located in the Dosso region, between 4° 1′ 40' East longitude and 13–38′ 40ʹ North latitude. The rainy season extends from June to October with an average annual rainfall between 600- and 800-mm. Malaria is meso-endemic with seasonal transmission during the rainy season. The most recent entomological studies indicated that the most common vectors are *Anopheles gambiae *sensu stricto (*s.s.*) and *Anopheles arabiensis* [18]*. Plasmodium falciparum* is the main malaria pathogen with a prevalence of 97% [19].

### Study population

The blood samples used in this study were collected from patients included in a therapeutic efficacy study comparing AL versus ASAQ, conducted in 2017, in Dogondoutchi. Patients included had a positive thick smear and uncomplicated *P. falciparum* malaria at the time of enrollment. Samples in this analysis (n = 159) included those from patients before treatment (n = 155), and therapeutic failures post treatment (n = 4). The four patient samples demonstrating therapeutic failure include two late clinical failures (LCFs) and two late parasitological failures (LPFs), after the 28-day follow-up period according to the WHO (2019) protocol.

Samples (n = 155) from the day of treatment (day 0) were sequenced at the *Centre National de Référence du Paludisme* (CNR), in Paris. The samples (n = 4) with therapeutic failure (day of failure) were sequenced at the University of Notre Dame, USA.

### Collection of blood samples

Both thick and thin blood smears, and malaria Rapid Diagnostic Tests (RDTs) were performed on patients using capillary blood taken by a finger prick. The SD Bioline Malaria Ag *Pf* (Standard Diagnostics Inc.) brand RDT was used to diagnose malaria positive patients towards inclusion in this study. Blood slides from RDT positive patients were transferred to the laboratory where the presence of malaria parasites was confirmed by standard microscopy. A dried blood spot was also collected from each patient by depositing a few drops of blood on Whatman paper (Whatman Grade GB003 / GE Healthcare). Dried blood spots were stored in individual plastic Ziploc bags containing silica gel desiccant. Samples were stored at room temperature before being transferred to the laboratory for DNA extraction and subsequent molecular analysis.

### DNA extraction and analysis of the multiplicity of infections

DNA was extracted from dried blood spots using four 1.4 mm diameter hole punches using the QIAamp DNA Mini kit (Qiagen, Manufacturer reference, 51306). Briefly, the samples were lysed with proteinase K followed by a series of washes with wash buffer solutions, binding of DNA to the membrane of a collector tube and finally the elution of DNA. The extracted DNA was stored at − 20 °C before polymerase chain polymerization reaction (PCR) [20].

**Fig. 1:**
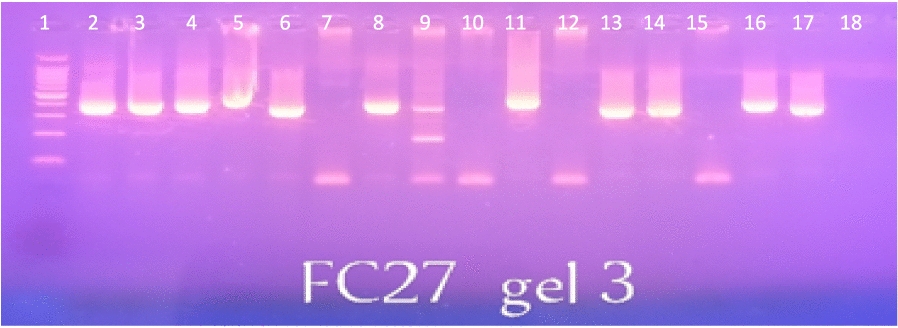
Agarose gel electrophoresis for *Pfmsp2/FC27*. Lane 1 = Molecular size ladder, Hyperladder IV (50-1013 kb, Bioline); Lanes 2, 4, 6, 8, 14 and 16 = *Msp2* allelic family FC27 at day 0; Lanes 3, 5, 9, 11, 13, and 17 = *Msp2* allelic family FC27 on day of treatment failure; Lanes 7, 10, 12, and 15 indicate negative samples; Lane 18 is a negative control without DNA

**Fig. 2:**
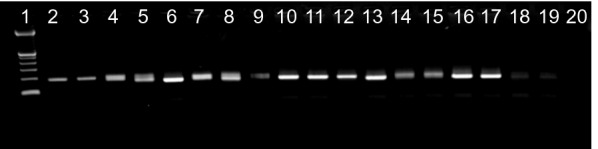
Gel electrophoresis for *Pfmsp1/K1*. Lane 1 = Molecular size ladder, Hyperladder IV (50-1013 kb, Bioline); Lanes 2, 4, 6, 8, 10, 12, 14,16, and 18 = *Msp1* allelic family K1 at day 0; Lanes 3, 5, 7, 9, 11, 13, 15, 17, and 19 = *Msp1* allelic family K1 on day of treatment failure; Lane 20 is a negative control without DNA

The distinction between recrudescence (True Therapeutic Failures) and reinfection (with a new mosquito bite) was analysed by nested PCR with markers for polymorphisms in *Pfmsp1* and *Pfmsp2* [21] (Figs. 1 and 2). This allowed for the analysis of multiplicity of infections (MOI) resulting in only monoclonal infections being sequenced (n = 155).

### PCR of k13 gene

**Fig. 3:**
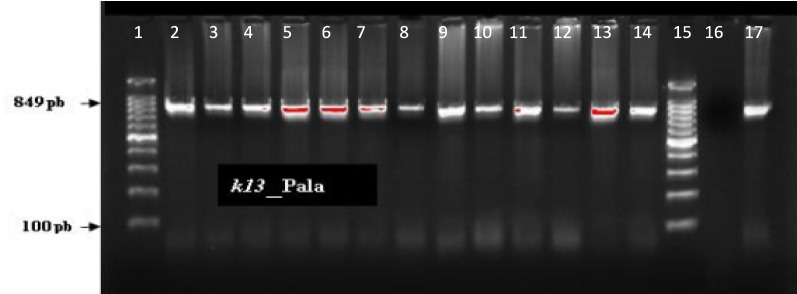
PCR2 agarose gel for amplication of *Pfkelch13* fragment. Nested PCR: Lane 1 = Molecular size Marker (Hyper ladder IV, 50–1013 bp, Bioline), Lane 2 -14 = band size of 849 pb, corresponding to the helix domain of the *Pfkelch13* gene, Lane 15 = Molecular Weigh Marker, Lane 16 is a negative control without DNA and Lane 17 = 3D7 control *Plasmodium* DNA

Amplification of the *k13* was performed using a nested PCR reaction. The first PCR (PCR1) used the following primers: K13_N1-F (CGGAGTGACCAAATCTGGGA) and K13_N1R (GGGAATCTGGTGGTAACAGC). The 20 µL PCR reaction mixture contained 2.5 µL of extracted DNA, 10 µM of each primer and 12.5 µL of the 2X PCR ToughMix reaction buffer (AccuStart II PCR ToughMix^®^, QuantaBio, 95142). The thermocycler conditions were as follows: 95 °C for 5 min, followed by 40 cycles of denaturation at 94 °C for 30 s, hybridization at 60 °C for 1’30 min, and an extension to 72 °C for 1’30 min; followed by a final extension to 72 °C for 10 min. The second PCR or Nested PCR (PCR2) used the following primers: K13-PCR_F (GCCAAGCTGCCATTCATTTG) and K13-PCR_R (GCCTTGTTGAAAGAAGCAGA). The 40 µL reaction mix contained 5µL of PCR1 amplification product, 10 µM of each primer and 25µL of the 2X PCR ToughMix reaction buffer (AccuStart II PCR ToughMix^®^, QuantaBio, 95,142). The thermocycler conditions were set as follows: initial denaturation at 95 °C for 15 min followed by 30 denaturation cycles at 95 °C for 30 s, hybridization at 58 °C for 2 min and an extension at 72 °C for 2 min followed by a final extension at 72 °C for 10 min. The amplified fragment (849 bp) was visualized after electrophoresis on a 1% agarose gel (Fig. 3).

### K13 gene sequencing

The PCR2 product (8 µL) was purified using enzymatic cleaning consisting of 2 units of exonuclease 1 (USB Corporation, Cleveland, OH), 1 unit of Shrimp Alkaline Phosphatase (USB Corporation) and 1.8 µL of bi-distilled H_2_O. This mixture was incubated at 37 °C for 15 min followed by another 15 min incubation at 80 °C to inactivate the enzymes. Each PCR product was sequenced at least twice from both directions using the two PCR primers to generate sense and anti-sense sequences with Sanger sequencing on the DNA analysis platform ABI3730xl (PE Applied Biosystems, Warrington, England). Any sequence with ambiguous base-pairs was re-sequenced.

### Analysis of the genetic variability of the k13 gene

**Fig. 4:**
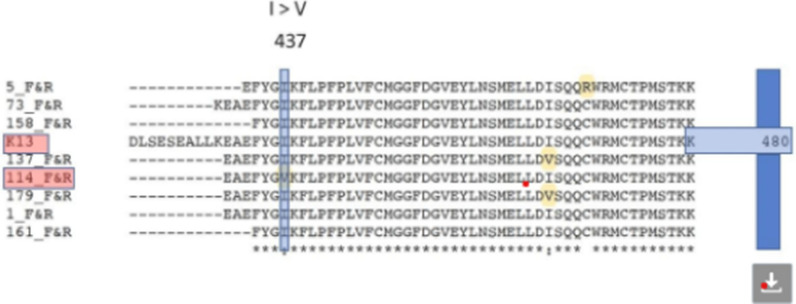
Comparison of field isolate sequences with the control sequence Pf_3D7

Forward and reverse sequences for each sample were aligned and single nucleotide polymorphism (SNP) mutations identified using the SeqMan Pro assembler (DNASTAR Inc., Madison, WI). The sequences were manually inspected for quality and re-sequenced when SNPs were ambiguous [20–22]. The translated DNA and protein sequences were compared to the wild-type *P. falciparum* Pf3D7 reference strain sequence (NCBI Reference Sequence: XM_001350122.1). Protein domains and motifs were identified through scans of *k13* wild type protein sequence using InterProScan 5 tool (Fig. 4) [23].

## Results

### Description of the study population

A total of 159 samples were sequenced for the *k13* gene associated with artemisinin resistance. One hundred and fifty-five (n = 155) samples were from patients at D0 (first day of treatment). Four samples came from patients with TF – of which two were LCFs and two were LPFs. The socio-demographic characteristics of the study population and treatment responses have been published [21].

### Polymorphisms in the k13 gene

One hundred and fifty-nine (n = 159) samples were sequenced. The success rate of the *k13* gene sequencing was 69.18% (110/159). Of the samples with successful sequences, 90% (99 of 110) were wildtype (WT) and 10% (11 of 110) had mutations. Ten different mutations were observed: C469R, T508S, A578S, I465V, R515T, I437V, L514L, F506L, P443P and P715P. Seven of these mutations were non-synonymous (C469R, T508S, R515T, A578S, I465V, I437V, and F506L) while three were synonymous (P443P, P715P and L514L). The prevalence of these mutations was 1.82% (2/110) each for T508S and I465V; and 0.91% (1/110) each for C469R, R515T, A578S, I437V, F506L, P443P, P715P, and L514L. Eleven samples displayed these 13 different mutations, with R515T and T508S mutations being the most common mutations (Table 1). These sequences are available in Genbank (Accession numbers: MZ364160, MZ364-213).

**Table 1:** Polymorphism of *Pfkelch13* gene in isolates from Niger

Mutation	Frequency	Point Mutation	Amino Acid	TTT	Statut	SNP
C469R	0.91	TGC-** CGC**	Cys^469^Arg	AL	LCF/ACPR	NSY
T508S	1.82	ACT-** AGT**	Thr^508^Ser	AL	LCF	NSY
R515T	1.82	AGA-** ACA**	Arg^515^Thr	AL	LCF/LPF	NSY
A578S	0.91	GCT-** TCT**	Ala^578^Ser	D0	ACPR	NSY
I465V	0.91	ATT-** GTT**	Ile^465^Val	AL	LPF	NSY
I437V	0.91	AGA-** ACA**	Arg^437^Thr	AL	LPF	NSY
F506L	0.91	TTT-CTT	PhF506Leu	D0	ACPR	NSY
P443P	0.91	CCA-** CCC**	Pro^443^Pro	AL	LPF	SY
P715P	0.91	CCC-CCA	Pro^715^Pro	D0	ACPR	SY
L514L	0.91	**TTA-TTG**	Ala^514^Ala	AL	LCF	SY
Wild	90					

It's a point mutation where a single nucleotide base is changed

### Analysis of the association between SNPs and treatment

**Fig. 5:**
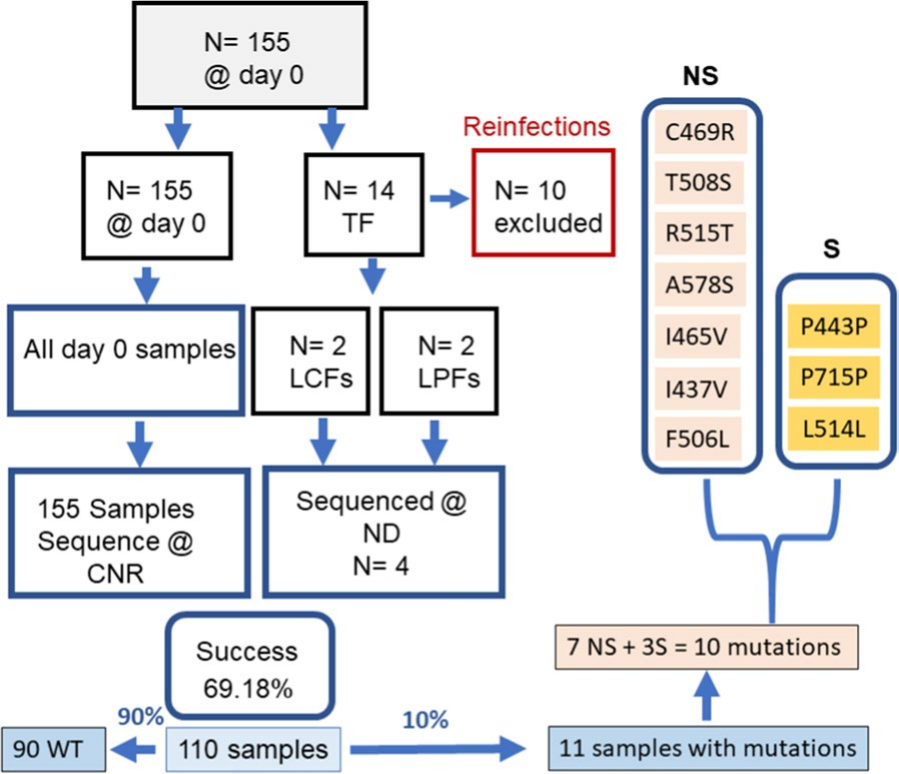
Graphical representation of the design of the study and the most critical findings. The number of samples collected at day 0 was 155. Only 14 samples among these 155 samples from day 0 showed a treatment failure (TF) during follow up. Ten (10) samples out the 14 TF were reinfections. All samples from day 0 as well as those with TF without reinfection were submitted for sequencing. LCFs (late clinical failures); LPFs (late parasitological failures); WT (Wildtype); NS (non-synonymous); S (synonymous); CNR (Centre National de Reference du Paludisme); ND (University Notre Dame)

Five mutations (C469R, F506L, N197D, H385H, P715P) were observed before the treatment (D0). From these five SNPs, two were non-synonymous (C469R, F506L,), and one was synonymous (P715P) (Fig. 5 and Table 1).

Seven mutations (C469R, T508S, R515T, L514L, P443P, I437V and I465V) were selected by AL of which five were non-synonymous (C469R, T508S, R515T, I437V and I465V) and two were synonymous (L514L and P443P). ASAQ did not select any mutation. The T508S and R515T mutation was selected twice (1.82%) by AL. These two mutations were considered the most selected by treatment (Table 1, Fig. 5).

### Analysis of the association between SNPS and therapeutic failure

Three non-synonymous mutations—R515T, I437V and I465V, were observed in patients with LPF. Five SNPs were observed in patients with LCF—C469R, T508S, R515T, L514L and P443P, of which three were non-synonymous (C469R, T508S and R515T) while two were synonymous (L514L and P443P) (Table 1).

### Recrudescence versus reinfection

Samples with treatment failure were assessed for multiplicity of infection (MOI). PCR and Nested PCR targeting *Pfmsp1* and *Pfmsp2* identified ten reinfections that were excluded from the analysis.

## Discussion

This descriptive and analytical study highlights polymorphisms in the *k13* gene in isolates from a randomized two-armed clinical trial comparing the efficacy of AL and ASAQ in Dogondoutchi, Niger. The study analyses selected mutations and anti-malarial treatment (AL or ASAQ) as well as the association between specific mutations and therapeutic failure (ACPR, LCF or LPF).

Sanger sequencing was used to sequence and analyse genetic variability of the *k13* gene. This method remains widely used for sequencing individual samples despite the advent of Next-Gene sequencing (NGS) and even nanopore or Minion [24] methodologies. PCR coupled with amplicon sequencing is an excellent method of genotyping *P. falciparum* strains.

The study of the polymorphism of the *k13* gene revealed 10 SNPs of which seven were non-synonymous (C469R, T508S, R515T, A578S, I465V, I437V, and F506L) and three were synonymous (P443P, P715P and L514L). With the exception of R515T and A578S [25], all these mutations are observed and described for the first time. They have never been reported by the World Health Organization (WHO) or the Worldwide Antimalarial Resistance Network (WWARN) [26]. Several studies have described polymorphisms of the *k13* gene, in Southeast Asia—in the Mekong delta, in Africa, and even in Niger [6, 27, 28].

On a global scale, the KARMA study was conducted on samples from five continents [23]. This study characterized high levels of polymorphism of the *k13* gene*,* with 108 non-synonymous point mutations being highlighted. The most important mutations related to resistance to ACT include C580Y, F446I, R539T, A578S, Y493H, P574L, P553L, N458Yand R561H [23]. In Southeast Asia, the epicentre of artemisinin resistance, four mutations were strongly associated with both slow parasitic clearances, and decreased in vitro sensitivity after culture adaptation of the ring stage survival assay (RSA) (Survival rate > 1). These mutations include Y493H, R539T, I543T and C580Y. Nine other mutations were weakly associated with artemisinin resistance and include P441L, F446I, G449A, N458Y, P553L, R561H, V568G, P574L and A675V [14]. In sub-Saharan South Africa, Kamau et al*.* observed 22 mutations, including seven non-synonymous—A557S, V566I, A569T, S576L, A578S, L589I, Y630F. However, this study did not assess the predictive value or phenotype of mutations [14]. In Niger, thirteen-point mutations have been described (M472I; Y558C; K563R; P570L; A578S; P615S; I465I; C469C; R471R; L488L; G496G; V510V and Y630Y) of which six are non-synonymous (M472I; Y558C; K563R; P570L; A578S; P615S) and seven are synonymous (I465I; C469C; R471R; L488L; G496G; V510V and Y630Y). Eight of these mutations were specific to Niger (M472I; Y558C; K563R; P570L; P615S; L488L; V510V and Y630Y) [16].

The prevalence of mutations observed in the study was often low (less than 1%)—being repeated once or twice in all samples. The KARMA study had only nine mutations with a prevalence greater than 1%, while in comparison, the prevalence of mutations in this study is 1.82%. This slightly higher prevalence in mutations may suggest an increase in the level of molecular resistance to ACT. The T508S and R515T mutations were selected twice by AL. In Mali, 26 SNPs were identified but F446I mutation was only seen twice [29]. Further investigations are required to validate them, at least in Niger.

Seven mutations (C469R, T508S, R515T, L514L, P443P, I437V and I465V) were selected by AL of which five were non-synonymous (C469R, T508S, R515T, I437V and I465V). Care was taken to ensure that SNPs determined were not artifacts of sequencing or miscalled bases, with each sample being sequenced from both directions twice. This study highlights the nature of artemisinin-based combinations in the selection of mutations in the *k13* gene. The mutations R515T and T508S are the most important since they are more frequent, therefore possibly under higher drug selection pressures. In a previous study analyzing the nature of SNPs before and after ACT treatment in Niger, the *PfK13A569G* non-synonymous mutation was selected by ASAQ, and associated with TF in Niger [16].

Few samples with multiple non-synonymous mutations in the K13 polymorphism have been reported. This study reveals two samples that have three (C469R / T508S / R515T) and two (R515T / I437V) non-synonymous SNPs, respectively. More research has to be conducted to see if artemisinin resistance has a cumulative effect, such as that seen in resistance to sulfadoxine-pyrimethamine.

Previous investigations used the mutagenesis technique (Z-Finger-Nucleases) coupled with the RSA technique and demonstrated that the R539T and I543T mutations are associated with a very high levels of artemisinin resistance [30]. On the other hand, the C580Y mutation is associated with variable resistance levels [30]. In contrast, a study conducted in Cameroon found no mutations associated with therapeutic failure [31].

C469R, T508S, R515T, L514L and P443P mutations were seen in patients with LCF. In contrast, R515T, I437V and I465V mutations were found in patients with LPF. Therefore, parasites present in patients with LPF and LCF were those carrying the mutations allowing them to survive drug pressure. These parasites can and may play a dominant epidemiological role if they become more widespread and responsible for the spread of artemisinin resistance. One of the driving concepts of ACT use is to mitigate the emergence and spread of parasite resistance by using a partner molecule that would require the emergence of resistance mechanisms to both molecules. This study demonstrates the role of parasites from LPF and LCF in the selection of resistance. In contrast, in Mali, though 26 SNPs were identified, none were observed in patients with therapeutic failure [29].

The limitations of the study include the absence of parasitic clearance time, phenotypic data and an analysis of polymorphisms in microsatellites adjacent to the *k13* gene to elucidate evolutionary mechanisms. The study of genetic polymorphism of strains using *Pfmsp1* and *Pfmsp2* markers confirms the absence of MOIs in the analysed samples. The reinfection samples that were excluded from the study were not sequenced. Whether some of these excluded samples had any *k13* mutations *is unknow*.

This data is essential to the National Malaria Control Programme (PNLP) and the Ministry of Public Health (MPH) in Niger, that monitors anti-malarial resistance and defines optimal strategies for fighting malaria in Niger. This study also demonstrates the emergence of novel and multiple possible resistance SNPs in the malaria parasite that may confound malaria elimination efforts.

It would be important to supplement this sequencing study with one that examines functional genomics—specifically to edit the R515T and T508S mutations using the CRISPER Cas9 or Z-Finger Nuclease techniques towards inferring functionality.

## Conclusion

The evidence generated by this study suggests that artemisinin-based combinations used for treatment of uncomplicated *P. falciparum* malaria, have selected novel *k13* polymorphisms in a 2017 clinical study in Niger. The sequencing reveals the simultaneous selection by AL of five non-synonymous SNPs. Data implies that the *k13* polymorphisms were selected in vivo (in host) by the drug treatment either de novo, or more likely, from polymorphisms present at very low levels prior to ACT treatment. The epidemiological impact of mutations R515T and T508S and their potential role in resistance to artemisinin must be confirmed by functional genomics or gene editing techniques. The results are highly important since they present cautionary evidence, appear to be the result of novel mutations in the *k13* gene, and the extensive post-treatment selection demonstrated has not been reported elsewhere in Africa, where artemisinin-based combinations remain largely efficacious. Continued monitoring of *k13* gene polymorphisms is vital towards understanding the emergence and spread of resistance to malaria drugs in Africa.

## Data Availability

Not applicable.
